# Root Tropisms: New Insights Leading the Growth Direction of the Hidden Half

**DOI:** 10.3390/plants10020220

**Published:** 2021-01-23

**Authors:** Luigi Gennaro Izzo, Giovanna Aronne

**Affiliations:** Department of Agricultural Sciences, University of Naples Federico II, Via Università 100, 80055 Portici, Italy; aronne@unina.it

**Keywords:** directional growth, tropistic stimuli, root bending, Cholodny–Went, statoliths, auxin, altered gravity, microgravity

## Abstract

Tropisms are essential responses of plants, orienting growth according to a wide range of stimuli. Recently, considerable attention has been paid to root tropisms, not only to improve cultivation systems, such as those developed for plant-based life support systems for future space programs, but also to increase the efficiency of root apparatus in water and nutrient uptake in crops on Earth. To date, the Cholodny–Went theory of differential auxin distribution remains the principal tropistic mechanism, but recent findings suggest that it is not generally applicable to all root tropisms, and new molecular pathways are under discussion. Therefore, an in-depth understanding of the mechanisms and functions underlying root tropisms is needed. Contributions to this special issue aimed to embrace reviews and research articles that deepen molecular, physiological, and anatomical processes orchestrating root tropisms from perception of the stimulus to bending. The new insights will help in elucidating plant–environment interactions, providing potential applications to improve plant growth on Earth and in space where microgravity diminishes or nullifies the gravitropism dominance.

## 1. Introduction

Plant development is largely influenced by tropisms which govern organ position and growth during the whole life cycle. Roots can explore the surrounding environment through various sensing mechanisms, reorienting growth towards or away from signal sources, including gravity, light, touch, water, chemicals, temperature, magnetic fields, electricity, oxygen, and sound [1]. Depending on the nature of the stimulus, several root tropisms have been identified which include gravitropism, phototropism, thigmotropism, hydrotropism, halotropism, chemotropism, thermotropism, magnetotropism, electrotropism, oxytropism, and phonotropism. Tropisms can be positive or negative according to the exhibited growth toward or away from the directional stimulus, respectively, and are exerted through differentially-regulated cell growth on opposite sides of the root tip that ultimately results in root bending [2]. In this regard, the term “tropism” must be carefully defined, considering that some stimuli can induce an escape response resulting from an inflicted damage, possibly related to the magnitude of the stimulus. Further investigation on chemotropism, electrotropism, thermotropism, oxytropism, and phonotropism will have to determine if these growth responses are indeed tropistic responses, and what are the underlying mechanisms.

As early as the late 1800s, Charles Darwin and his son understood that the root tip has the power to direct plant movements [3]. To date, thanks to studies carried out on model species such as *Arabidopsis thaliana*, four zones in the root tip have been identified, namely the meristematic zone, the transition zone, the elongation zone, and the differentiation zone, each characterized by specific cell types, cellular activities, and responses to tropistic signals [4]. The perception sensors of the various tropistic stimuli vary notably from starch-filled amyloplasts (gravitropism) to phytochromes and phototropins (phototropism), but for most tropisms the sensors, or even the sites of perception and action, are still unidentified [1]. In addition, the signal perception sensors can act in multiple tropisms, as in the case of phytochrome, which is involved in the regulation of both gravitropism and phototropism of roots [5]. Indeed, between sensing and bending, signal transduction pathways are partly shared among tropisms, and further research is needed to assess similarities and differences between tropistic responses. In the last few decades, several studies have been conducted with a focus on the molecular mechanisms underlying tropistic responses, enabled by advancements in molecular genetics and supported by special research environments such as the International Space Station (ISS) [6]. Currently, research on plant tropisms is critical for improving plant-based life support systems in space, considering that a more in-depth knowledge is required to guide root growth in altered gravity, especially microgravity. At the same time, the increasing possibility of performing experiments in space, together with the progress of technology, such as the implementation of light emitting diodes (LEDs) in the ISS laboratories, is paving the way to a better understanding of plant tropisms, aiming at improving plant production in space [7].

Even though plant movements have long fascinated scientists around the world, the mechanisms that control the different root tropisms are yet to be elucidated, and recent years are providing numerous opportunities for tropism research thanks to scientific and technological progresses. Hence, we are pleased to present this special issue of *Plants* that centers on tropistic responses to the various stimuli governing root growth and orientation. The aim is to push forward our understanding of plant–environment interactions, gathering original research and review articles that deepen molecular, physiological, and anatomical processes orchestrating root tropisms from perception of the stimulus to bending.

## 2. Highlights of the Special Issue

### 2.1. Sensors, Signalling, Action Regions, Phytohormones, and Secondary Messengers

Many molecular aspects of some root tropisms have been understood, especially for gravitropism, phototropism, and hydrotropism. Tropism research has benefited greatly from advances in molecular biology and the use of model species such as *Arabidopsis thaliana* to characterize sensors and signaling pathways [1]. Still, there is a shortage of knowledge for most root tropisms, and even some sensing mechanisms remain unknown. An open issue is the capacity of plant roots to sense electric fields and sounds mediating electrotropic and phonotropic responses respectively, and future studies must shed light on these phenomena.

Although the Cholodny–Went theory of differential auxin distribution remains the principal tropistic mechanism after almost a hundred years from its formulation, recent studies suggest that this theory is not generally applicable to all root tropisms, as in the case of root hydrotropism [8]. Hence, further studies are necessary to refine the theory and to explore commonalities and differences in the molecular and physiological processes that orchestrate root tropisms, including sensors, signalling pathways, action regions, phytohormones, and secondary messengers. Currently, the discussion also involves abscisic and gibberellic acid, brassinosteroids, cytokinins, ethylene, flavonoids, calcium, and reactive oxygen species as part of a dynamic system of sensing and signalling which mediates root bending [1]. Furthermore, there is a pronounced difference in tropistic responses among plant species, as shown for root phototropism almost a century ago by Hubert and Funke [9], but this has been largely overlooked for most tropisms. Systematic investigations will provide a broader understanding of such phenomena, giving new insights to elucidate evolutionary patterns and ecological significance of tropisms in plants.

### 2.2. Experimental Tools and Environments

Recent years offer increasing chances to deepen our understanding of plant tropisms through advanced scientific tools and environments. To better characterize root tropisms, there is a need to study single tropisms without confounding effects of other tropistic stimuli. For this purpose, there is a growing potential for applications of new technologies in tropism research, and specific devices have already been developed. An example is the “D-root” system which allows tropism research, preventing confounding effects of light-reaching roots, which is known to trigger phototropic and growth responses [10]. Furthermore, numerous studies on root tropisms have been performed by altering the gravity stimulus to reduce the dominance of gravitropism, which typically masks other tropisms. Indeed, specific devices such as clinostats, random positioning machines (RPMs), and centrifuges, but also parabolic flights, drop towers, rockets, and magnetic levitation provide opportunities to explore plant tropisms in altered gravity. More specifically, clinostats and RPMs regularly change the direction of gravity perceived by the plant, whereas microgravity in space can be best described as weightlessness achieved by the balance between the gravity force and the centrifugal force of the object, which is also the case of parabolic flights and drop towers [11]. In this framework, although studies on Earth using devices which change the direction of gravity proved to be informative for certain parameters, findings necessitate space-based research to be validated [11]. Currently, the possibility to perform experiments in space using laboratories onboard the ISS is gaining much interest in tropism research, especially for root tropisms, which are dominated by gravitropism [12]. Future studies on mechanisms and regulation of root tropisms will benefit strongly from weightlessness environments where gravitropism can be effectively eliminated, enabling more detailed studies on weaker tropisms. Research on plant tropisms is also indispensable for future programs in the framework of colonizing extraterrestrial environments. In this endeavor, an in-depth understanding of plant tropisms and their changes in altered gravity is needed to guide plant growth in space.

### 2.3. Tropism Interactions

Although it is fundamental to isolate tropistic stimuli to characterize each tropism, an in-depth understanding of tropism interactions is indispensable for applications in natural and controlled environments where the various stimuli can be perceived as tropistic signals by the plant. Tropisms interact in shaping plant development with a greater or lesser dominance with respect to the others, but the underlying relations between tropistic stimuli and their magnitude need to be further investigated [13]. Indeed, the magnitude of each stimulus can determine the final root bending, and the response can be sometimes unpredictable, as in the case of the positive phototropism of *Arabidopsis* roots to blue light in microgravity [14]. The knowledge regarding phototropic responses of roots already contributes to the improvement of cultivation systems in controlled environments, such as those in space, but other stimuli have also shown potential for applications, as in the case of root chemotropism [15]. Still, the question remains whether tropisms other than gravitropism play a substantial role in determining root growth direction in a natural setting on Earth, where roots are subjected to several stimuli simultaneously. In this regard, important requirements include knowledge about the relative strength between tropisms and their masking thresholds. For instance, the vector hypothesis has been proposed to describe the actual degree of root bending depending on the phototropic response and a counteracting gravitropic response of roots [16,17]. However, more studies are necessary to further verify the phenomenon and evaluate tropism interactions at different levels of stimulus magnitude. Understanding the role of the different tropisms in nature and their relative strength in governing plant development would help improve plant cultivation strategies to maximize resource use efficiency. Despite that several studies have investigated root tropisms and their interactions, knowledge about the mechanisms involved is far from complete, and further research is needed to shed light on the actors involved and their functions.

## Data Availability

Not applicable.
